# Examining the power of the social imaginary through competing narratives of data ownership in health research

**DOI:** 10.1093/jlb/lsaa068

**Published:** 2021-02-04

**Authors:** Annie Sorbie, Wifak Gueddana, Graeme Laurie, David Townend

**Affiliations:** 1 Edinburgh Law School, Mason Institute for Medicine, Life Sciences and the Law, Edinburgh University, UK; 2 Department of Digital Humanities, Kings College London, UK; 3 Department of Health, Ethics, and Society, Care and Public Health Research Institute (CAPHRI), Maastricht University, NL

**Keywords:** barriers, data sharing, ownership, property, stewardship

## Abstract

This article explores the social imaginary in the context of data ownership and the (non-)delivery of the data sharing revolution in biomedicine. We contribute to this special issue on imaginaries by developing a method and paradigm of ‘competing narratives’. Despite multiple initiatives to encourage health data sharing, and a strong ‘open access’ agenda, the data sharing revolution is not yet delivered. Ownership is persistently (though inconsistently) presented as a barrier to data sharing. However, existing literature does not reveal how far appeals to ownership are part of the problem. This paper reports original, interdisciplinary research asking: in health research, in what ways, if at all, do notions of ownership (broadly conceived) of health-related data impact on sharing practices? Doctrinal and empirical research methods are used to expose evidence of drivers behind appeals to ownership in health data sharing. The findings speak to how funders and data custodians can better tailor existing and potential data sharing initiatives to perspectives and behaviors. The concept of ‘my data’ is important: notions of reward, opportunity, control, and safeguarding establish legitimate, potentially competing ‘ownership’ interests in data. In particular, this research raises questions about the long-term effectiveness of an open access ideology that ignores these subtleties. In conclusion we find power in the social imaginary of ownership with respect to biomedical data; however, that power emerges and is enacted in unexpected ways by multiple actors within the ecosystem, often driven by competing narratives about what is at stake. Importantly, formal legal property-type appeals to ownership appear to have far less power in the narratives about data than the ethical and social concerns that underpin responsible biomedical research.

## I. INTRODUCTION: DELIVERING THE DATA SHARING REVOLUTION?

The benefits of effective data sharing and (re)use are well acknowledged.^1^ These are both generic—the potential for science to be advanced effectively through the optimization of existing data sets^2^—and also health specific—as clinicians strive to translate research findings into clinical practice.^3^ The economic and scientific case for sharing research data^4^ is recognized by funders^5^ and promoted in publications such as the UK Concordant on Open Data Research.^6^ Furthermore, a PLOS ONE study indicates that most clinical and scientific researchers working with biomedical data appreciate that data sharing and reuse are important to their work.^7^

Despite this momentum in data sharing, those who work with health data have reported that the ‘data sharing revolution’^8^ has not yet been delivered. Evidence further suggests that practices of data sharing are uneven among existing health constituencies. For example, Pisani et al. indicate that the data sharing practices of epidemiologists and public health researchers may lag many years behind those researchers working in the field of genomics.^9^ Even here, Knoppers et al. recognize that while data sharing in genomics has been accelerated by the Human Genome Project there are still ‘inefficiencies and insufficiencies’ to be addressed.^10^

‘Data sharing’, both in this paper and more generally, is the practice of allowing access to personal data between different researchers. This immediately poses the question, ‘access on which and whose terms?’ And this question is at the heart of the research on which this paper is based. In all fields of research and commercial activity, where commodities of value are traded and exchanged, there is a system of control—often called ‘ownership’ or ‘property’—with rules and conventions (legal and more informal) about the extent to which the commodity that has been created by one individual or institution can be used by another. At one level, this model appears also to operate for personal data that are regulated, internationally, between persons from whom data are initially gained and the person(s) so gaining the data. The question of how far one individual can pre- or proscribe the access and use of personal data by another is extremely live and complex. But a more fundamental question is whether this is an example of ‘property’ or ‘ownership’ in action? As a matter of human dignity and human rights, the patient explaining their condition to a doctor or giving a blood sample cannot expect to be paid, at least in most jurisdictions, for their personal data, but they can expect that it is protected as a matter of human rights law relating particularly to their privacy. Indeed, most jurisdictions eschew any sense that there is formal, legal property in ‘raw’ data that might make the transaction of gathering the data in the first place a transaction where value might attach. However, there is also a competing tradition in most jurisdictions, relating to the creation of transferable commodities through human intellect, and that is most certainly an engine that generates ‘intellectual property’ (IP) that can be commodified for financial gain. This second tradition, in relation to the use of personal data between those who generate and then further process these data is certainly a matter of commercial interest. These are jurisdictionally specific rights expressing some internationally agreed concepts of ‘property’ and ‘IP’. Underlying these legal concepts, moreover, are broader concepts that have a more colloquial meaning. Thus, ‘ownership’ and ‘donation’, even ‘sharing’ and ‘giving’, are grounded in the relationship between people about things, that do not have necessary legal meanings, but are the informal terms that are used and which regulate how those relationships work. These appeals to property-like claims imply some degree of ‘control’, perhaps even fairness about expectations of what might be done on the basis of the relationship, but they are informal, and unenforceable; they are social, linguistic hints of belonging and determination. Notwithstanding they express expectations (perhaps even of fairness), that they are still used suggests an unresolved tension between the law that rejects a formal ‘property’ in some cases, and the colloquial ‘these data are mine’. This paper does not seek to limit the definitions of these terms, because of that social, linguistic plurality of meaning—the social imaginary of a plethora of terms implying the ability to pre- or proscribe access to the ‘thing’ or personal data, without the clarity and enforceability of law. Rather it seeks to report research that explores the extents of that imaginary amongst the ever-growing community of those who are engaged in the generation and analysis of personal data for biomedical research.

The impetus for this research is the *DataTerms* project that emerged from the experiences of Wellcome as a funder that, in discussions around the use, reuse and sharing of health data in research, terminology was frequently used in relation to the ‘ownership’ of data sets.^11^ Anecdotally this was in the context of ownership acting as a barrier to sharing, and serving to restrict access. A 2017 joint report by the British Academy and the Royal Society on data management and use^12^ captures the changing relationship in society, and by extension, governance:

‘The traditional data lifecycle was clear, relatively sequential, predictable, often managed by a single organisation and made it comparatively easy to erase data sets that were no longer needed. These characteristics also meant that data governance could focus on a specific point in the ‘cycle’, such as on collection, and use this single point to gain significant leverage over the broader process. However, this traditional approach is now under considerable strain.’^13^

The report suggests that, in this shift from near linear data usage to multi-directional, networked relationships to data, the meanings of key legal and governance concepts, including privacy, ownership and consent, have shifted and continue to do so, and that ‘ownership’ is a concept under ‘unprecedented strain’ and giving rise to ‘significant tensions’.^14^ This focus on ownership was of particular interest to the *DataTerms* team (composed of three academic lawyers and a social scientist) as the legal terminology that is provided by data protection legislation—as the main legal framework governing data sharing and use—is simply not couched in these terms. This disjunct between law and community perspectives suggested that appeals to ownership were about more than attempts to stake legal claims to data. We explore the legal landscape further in Section II below.

In this paper we do not seek to argue for or against a property-based system of data sharing. Rather, the *DataTerms* project explored current experiences and practices around data sharing in order to better understand whether ownership is indeed perceived to be an obstacle and, if so, to expose any evidence behind these appeals to ownership, whether these are in a strict legal sense or in a subtler metaphorical or philosophical sense.

In framing the contribution of our research, we are not suggesting that shortcomings in delivering the data sharing agenda have been ignored or neglected. Indeed recent initiatives across research communities have advocated for a human rights approach to an international code of conduct for genomic and clinical data sharing,^15^ a global privacy governance framework directed to establishing privacy law harmonization for biobanking,^16^ a standardized format for citing (and thereby incentivizing) data generators,^17^ and the adoption of principles on the legal interoperability of research data.^18^ However, while the general aspiration of data sharing is broadly seen as a ‘good thing’, existing evidence, as gathered by the Royal Society, indicates that actual behaviors and practices vary significantly across scientific sectors, research ecosystems, and other techno-socio domains.^19^

Taken together, this body of literature and initiatives suggest a disconnect between ‘top-down’ policy and ‘bottom-up’ practice in health data sharing. This divide seems likely to become more, rather than less, pronounced as new health data sharing initiatives, such as data philanthropy,^20^ rely not only upon novel data flows from those who donate data altruistically, but also seek to maximize the health and social benefits derived from that data by effective sharing more widely within and beyond the (health) research community.^21^

Our core proposition is that, if stakeholders in health research are to tackle barriers to the reuse and sharing of health data effectively, and to implement existing and potential data sharing initiatives fully, then we must first seek to understand better the attitudes and the narratives that underpin these obstacles; that is, what assumptions, expectations, and stories underpin the language of ‘my data’? ^22^ Thus, while barriers to effective data sharing and reuse are undoubtedly multi-faceted and context specific, in this study we focus our contribution on one aspect of this bigger picture: how notions of ownership (broadly conceived) of health-related data impact upon effective data sharing.

The paper starts with a brief analysis of the role that ownership plays in health data sharing, with reference first to the law, and then to wider motivational barriers (Section II). This highlights that while explicit appeals to ownership, in a narrow legal sense, are persistent, it is not clear how these are collectively understood by multiple stakeholders who interact with data in different ways. We find that ownership can be characterized in multiple and competing ways, both within and beyond the law. In Section III we go on to describe how this informed the mixed-method design of our empirical enquiries, which comprised 54 completed questionnaires, three focus groups and two interviews. Here we introduce the idea of ‘competing narratives’, both as a phenomenon and as a method, which helps us to understand how users interact with data and the narratives that they construct around this. Our findings, in Section IV, suggest that quantitatively the construct of ownership per se has low significance. However, the results of the qualitative analysis show multiple narratives and a nuanced understanding of ownership in a broader sense. In Section V we discuss these findings, thus bridging a gap between the existing literature on data sharing barriers and that on initiatives to address such barriers. In our conclusion we find that there is indeed much power in the social imaginary of ownership with respect to data in the biomedical sphere; however, that power emerges and is enacted in unexpected ways by multiple actors within the ecosystem, often in ways driven by competing narratives about what is at stake. Importantly, formal legal property-type appeals to ownership appear to have far less power in the stories that are told about data than the ethical and social concerns that underpin responsible biomedical research.

## II. OWNERSHIP AND HEALTH DATA SHARING: LEGAL AND MOTIVATIONAL BARRIERS

The law provides neither a straightforward nor comprehensive account of the role that ownership plays in health data sharing. In this section we briefly outline why this is so, before widening our consideration to how motivational barriers to data sharing may provide a more holistic account of appeals to ownership. We then synthesize these findings to draw out the implications for our empirical enquiries.

While the law, per se, is consistently identified as a potential barrier to effective data sharing,^23^ references to the role of ownership in this context are persistent, yet also patchy and under-developed. By way of example, Panhuis et al.’s 2014 systematic review of barriers to data sharing in public health^24^ includes factors such as the ‘guardianship or ownership’^25^ role adopted by those who collect data, which could result in a default position of restricting access to data, and ‘copyright’^26^ being used to restrict data access. Alongside this, the authors point to uncertainties and inconsistencies around who ‘owns’ public health data, as well as anxieties relating to the ‘protection of privacy’.^27^ In their Chatham House Report of 2015, Sane and Edelstein revisit the legal barriers identified by Panhuis et al.^28^ and identify ‘strict personal data protection laws’^29^ and the associated ‘ambiguous legal framework’ as the main barrier to data sharing, making only brief reference to ‘intellectual property rights and data ownership’,^30^ amongst other relevant matters.

Indeed, the reality is that those engaged in health research may well approach the legalities of using and sharing data with some trepidation—there is no single source of law that governs this endeavor. These challenges are multiplied when sharing occurs between countries or regions, and so across diverse legal systems and data sharing cultures. However, as noted at the outset, data protection legislation in Europe does not describe the relationships that stakeholders have with data in terms of legal ownership,^31^ nor has it ever done so. Similarly, where a common law duty of confidentiality arises, with the effect that identifiable health-related data cannot normally be disclosed without an actionable breach arising (save in limited circumstances, including where consent has been obtained or disclosure is in the public interest), the position remains that these relationships do not operate in formal terms of ownership. Rather, obligations are owed on the basis of a privacy or confidentiality framing of the ‘how’, ‘when’, ‘where’ and ‘why’ personal data or information are processed.^32^

Looking more widely, IP law—the protection of  ‘creations of the mind’ by, most commonly in health research, patents, copyright or trademarks^33^—is a related legal domain that has also been closely linked to data sharing.^34^ The impact of intellectual property rights (IPRs) on health data sharing has been considered by Andanda in the context of clinical trial data. She identifies that: ‘…a worrying trend has emerged whereby researchers often cite IPRs as a reason why results cannot be disseminated’.^35^ She suggests that ‘[t]he unwillingness to share data freely certainly means that some researchers consider their data proprietary “with a competitive advantage over other groups in terms of discovery and further acquisition of funds that would expand their research operations”’.^36^ This implicates notions of ownership both in a legal sense, but also in terms of researchers’ proprietary approach towards data as an exclusionary activity that militates against access and sharing. Andanda’s characterization of this as a ‘worrying trend’ suggests that, in the circumstances she describes, this is not a ‘good’ reason to restrict access to data.

More recently, in October 2016, the CODATA Legal Interoperability Interest Group reported on the Legal Interoperability of Research Data: Principles and Implementation Guidelines (the CODATA Report).^37^ This report considers the ability of researchers (generally, as opposed to in health research specifically) to reuse, share and access data for research purposes in the context of IP law.^38^ The premise of the CODATA Report is that there is a lack of understanding and information around legal issues concerning research data generally. The report gives examples of how IP law can impede data sharing where there is a lack of clarity around the legal conditions under which data may be reused,^39^ both on the part of those who disseminate^40^ as well as use research data.^41^

This suggests yet another way in which notions of ownership of (health) data might impede data sharing, namely, in terms of the (actual or perceived) complexities of the IP regime resulting in a default position where access to data is restricted.^42^ This analysis aligns with a key challenge faced by data linkage initiatives, where a complex legal landscape (largely, but not exclusively, in data protection terms) can lead to a ‘culture of caution’. Sethi and Laurie^43^ highlight that this is typified in contexts where stakeholders—be they researchers or regulators—default to a conservative approach, with the effect that a margin of conservative interpretation of law is adopted that drives behaviors at levels well below what the law actually requires or allows. A further compounding feature of law in both the spheres of data protection and IP law is that law rarely, if ever, *mandates* sharing. Compulsory IP licenses are notoriously contentious, and data protection law tacitly favors the non-processing of data over processing. In short, all of this is indicative of a culture where doing nothing *is* an option.

Lawyers are often inclined to view what we see as legal terminology—such as ‘ownership’—with some rigidity, but it is not uncommon for ‘property’-based language to be used in relation to confidential information in a ‘loose, metaphorical sense’.^44^ As highlighted by Montgomery,^45^ as individuals’ data are tested and analyzed—and, in the case of health research, anonymized and aggregated—this can give rise to multiple and potentially conflicting proprietary claims to ‘my data’ from the individuals themselves, clinicians, health researchers and even institutions. This suggests that creating ‘value’ *from* data may be quite distinct from the recognition of inherent value *in* the original data, and is an instance of how competing narratives around data sharing may be constructed by multiple stakeholders. Thus, the citizen who talks of ‘my data’ refers to information that relates to her in some significant and/or profound way (e.g. because a lack of control might compromise privacy or confidentiality), while others talk of ‘my data’ as a reflection of the labor spent to create and curate a dataset. In this way the motivations behind the loose, metaphorical appeals to ‘my data’ may be quite different. Put otherwise, there might be much more meaning hidden beneath apparent appeals to ownership than a mere desire for a property-based claim to control data akin to other forms of tangible property, like a car or a cello.

These observations inform our empirical enquiries in a number of ways. First, while both data protection and IP law may be engaged by health data sharing, the effect of these legal frameworks—in terms of how they are understood by stakeholders and impact upon data sharing practices—is far from clear. In this sense the law alone cannot account for the persistence of notions of ownership in relation to health data, and a more holistic approach is demanded. The analysis above has, however, underlined three more general points about the legal landscape.

The first observation is that ownership often appears alongside concerns relating to privacy and data protection. The proximate consideration of these last two concepts as key legal barriers to sharing health data is, perhaps, unsurprising, given that functionally they can perform similar exclusionary roles of non-access as do appeals to property or ownership. But as a matter of strict law, a property paradigm is very distinct from a privacy or data protection paradigm; failure to appreciate this suggests that how barriers to data sharing are labeled may have the potential to obscure the core concerns that underpin (non)sharing behaviors. Ownership with a basis in reward for added value arising from IP, and the human right to privacy are certainly also barriers, but seem also to be demanding questions of the presumptions of open access data sharing. In other words, how can respect for IP and human rights be reconciled with open access agendas?

The second point relates to the complexity of the legal landscape more generally, and the culture of caution this can drive, potentially giving rise to an environment where doing nothing *is* an option. There is a need to understand better how this complexity is perceived and navigated.

Third, there is some evidence of a degree of ambivalence towards notions of ownership in relation to data—for example in the description of the invocation of IPRs as a ‘worrying trend’. This is subtly different from the position in relation to data protection law, where, broadly, it seems to us that while there may be anxiety about ensuring that data subjects are protected, there is a broader acceptance of the ‘good’ in securing this protection in some form or another. In other words, appeals to ownership might be cast both in a positive light (as a proxy for privacy protection) and in a negative light (as a worrying attempt to control and restrict use): motivation, then, is all important.

Further to this, Knoppers et al. suggest that research may be hindered by worries around the sharing of benefits, such as scientific recognition, where researchers have invested time and effort in their work, particularly in the context of research groups in low-income countries.^46^ Factors identified that may heighten these concerns include the lack of incentive to share and a highly commercially and academically competitive environment. Similarly, when considering motivational barriers, Panhuis et al. also point to a lack of incentive to share data (for example, where these is little scientific credit to be had), the opportunity-cost of investing time and effort in data sharing where the value may be extracted by others (again, resulting in a loss of scientific credit and/or opportunity by the original data curator), and the possibility that shared data may be criticized or put to an unwanted or inappropriate secondary use.^47^ In their 2015 study of the motivations and barriers to biomedical data sharing, Federer et al. surveyed researchers’ attitudes towards and experiences of data reuse and sharing.^48^ They found that ‘sharing research data is a complex issue presenting many challenges’,^49^ and highlight similar barriers to those outlined above. As well as technological barriers—such as the knowledge and experience of using data repositories—they note that ‘special’ issues may apply where there are concerns about the identification of data subjects.^50^ Indeed, where clinical researchers indicated they had not shared data, the most common reason given for this was concern for research subjects’ privacy.^51^ However, like Panhuis and Knoppers, Federer et al. also note other matters that may impact on researchers’ willingness to share data, which could be considered to be more aligned with researchers’ proprietary claims. These include concerns that a third party might publish on the basis of a data set first, or without credit, or that shared data might be questioned, misused or misinterpreted.^52^

All of this suggests that to understand better how ownership in the context of these complex interwoven legal landscapes is understood and deployed by those engaged in the data sharing endeavor, more research is required on drivers of behavior that are motivational, rather than purely legal. In particular, we find that ownership can be characterized in multiple and competing ways, both within and beyond the law. In the section that follows, we set out how this finding informed our approach and methodology to our empirical work.

## III. METHODS: STORIES OF EXPERIENCE AND NARRATIVE INQUIRY

Our aim in this exploratory empirical work was not to homogenize the multiple understandings of ownership, as identified above, and impose them on participants, but rather the opposite: to tease these apart in order to elucidate participants’ understandings of these constructs and relationships.^53^ This approach subscribes to a tradition of qualitative narrative inquiry^54^ and is intended to generate depth of understanding about competing views. Given the broad understanding of imaginaries adopted in this special issue, including as the shared (or, in our case, divergent) views of stakeholders on the hopes, promises and expectations to be engendered through technoscientific development, we propose that this is one way that these views can be elucidated in a more concrete way.

More specifically, competing views of narrative^55^ or competing narratives^56^ are a paradigmatic mode in which experience is shared and that experience itself is storied.^57^ In other words, telling these narratives allows us both to acknowledge that they co-exist as well as to capture how they are described. In the context of the current enquiry, we understand data users—be they researchers, curators, policy makers or data holders—to be storytellers and characters in their own and in one another’s stories. By collecting their views, we shed light on and expose the limitations, anxieties and socio-political processes through which key constructs are interpreted, practiced, corroborated and refuted. The use of this method within legal, health and technology studies increasingly is a way to demarcate political discourse (or the official vision) on (in this case) sharing data, from the lived experience of participants. This in turn allows us to construct a more accurate and bottom-up account of the challenges and benefits of implementing such a vision.^58^

Our fieldwork, which was carried out between Summer 2016 and Autumn 2017 (pilot, questionnaire, focus groups and interviews), engaged with professionals undertaking a wide range of health-related data sharing initiatives, and asked them where they saw barriers and limits on data sharing more generally, and how they discuss those barriers and limits. We conducted this research in two stages. First, we circulated a questionnaire, asking participants to rank possible barriers to data sharing in order to measure their perception of which are more and less significant. Second, we created a space for discussion, through conducting focus groups and interviews, where we asked participants to elaborate on the reasons for the ranking and give us examples based on their roles, affiliation, and everyday practices. Our aim in using a questionnaire was not to test a developed theory. Rather we used it as a synoptic device to bring together the constructs surveyed in the literature, and to allow participants to engage with them. We left free text space for open questions and interpretations, in order to generate text for open coding. This process is further elaborated below.

Our questionnaire was developed during a pilot stage, following discussions within the research team, and with Wellcome’s Expert Advisory Group on Data Access (EAGDA). In the final version, which was sent to potential respondents, Section 1 asked participants about their affiliation, roles and data uses. In particular, we asked participants questions about their work, roles and the settings or organizations in which they operate. This included specific questions about participants’ relationships to health-related data and the type of responsibilities/demands on data they have. In Section 2 we presented participants with 10 constructs and possible barriers to data sharing that had been identified in the course of our legal and literature review, namely: control, privacy, consent, cost, risk of data misuse, protection of others, protection of data subjects, IP rights, the legal landscape, and data ownership. We first asked participants about how significant they considered these to be, when they made decisions about holding and sharing health-related data, and then about how far—if at all—these changed in relation to their experiences of other’s practices around holding and sharing data, i.e. when they themselves sought access to data. Participants were asked to order and rank these on a five-degree-significance scale (from very high to not at all). Alongside the significance scale, we asked respondents to explain their choices and motives when ranking constructs. This was a free-text space for further reflection on the concept-relationships ranked as being the most and the least significant, and for participants to explain what they do to enforce or mitigate them, with some further prompts about the data sharing environment (for example, the role of ethics committees). The object was to draw out the relationships between the concepts identified, some of which related to *ownership* in a narrow ‘legal’ sense and others which were engaged by the holistic notion of ownership as explored in the context of our literature review (for example, motivational and attitudinal concerns). The inclusion of the free text explanation was designed to allow respondents to indicate something of their own conceptualization of the elements that we had presented. The third and final section of the questionnaire was open-ended, and designed to gather loosely-coupled opinions (not linked to the significance scale) on what might or might not constitute a barrier to data sharing.

The second stage of our fieldwork involved recruiting volunteers among the respondents for further participation in the project by way of focus groups and interviews. Outputs from these were added to the text data corpus for analysis. We expected participants to form a perception of what it takes to ensure sharing health data on the basis of their practices, i.e. affiliations, roles and their daily data uses. Therefore, the focus groups and interviews allowed participants to share experience and explain their personal interpretation of the benefits and challenges of sharing data. The focus groups were conducted online using ‘Blackboard’ webinar software, in order to facilitate international interactions across health ecosystems.

Our initial recruitment strategy focused on different areas within which data sharing occurred. These ‘ecosystems’ were roughly identified as: genomics, registries, longitudinal, regulatory, non-medical, and international. Networks of actors were identified within each ecosystem, and invitations were sent to members of networks (through the network manager) with links to the questionnaire (which was created on-line through ‘Qualtrics’). Respondents were invited to pass on the link to others they believed to be interested within their ecosystem (a ‘snowballing’ technique). Across the ecosystems, 54 completed questionnaires were logged in Qualtrics and included within the analysis in this paper. Three focus groups were subsequently conducted with between 2 and 3 participants each, as well as two one-to-one interviews.

The participants had various (and sometimes multiple) roles (Table 1), across a wide range of health and health-related institutions (Table 2) in metropolitan cities in the UK, Europe, America and Asia. Some 22% of the participants were not using data directly and were coded as health professionals who take part in review committees and the practice of health research more generally. This diversity of roles and institutes is indicative of the complexity and multiplicity of the health research constituency. It also shows how healthcare research and delivery are intermingled; the majority of participants were based in university hospitals, public health institutions, and non-profit private charities (68% of the total sample). These participants had access to patients’ data and primary and secondary electronic health records, while using health data for a variety of purposes across the wider health community.

**Table 1:** Roles of participants

Consultant	1
Curator	19
Ethics Committee Member	16
Healthcare Professional	13
Member of Data and Sample Access Committee	1
Research Administrator	1
Researcher	39

**Table 2:** Affiliations

Charities and Private Research Trusts	4
National Commission, Health Department Funding	3
Information Governance Committees, NHS Wales Informatics Service, NHS Digital and other Health Informatics	8
Registries and Biorepositories	6
Universities, Hospitals and Non-profit Public Institutions	46

## IV. FINDINGS

### IV.A. Perceived Significance of Barriers to Data Sharing

**Figure 1:**
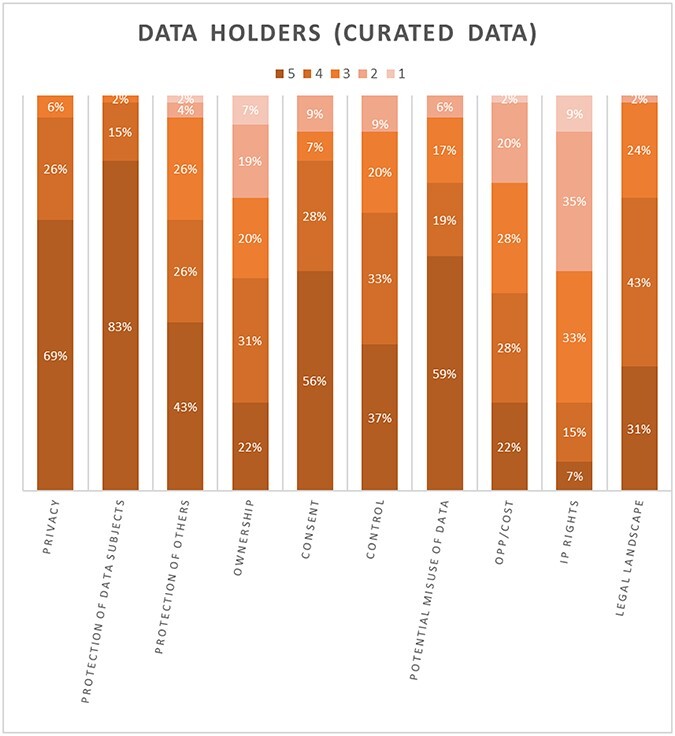
Perceived Significance (Data holder)

**Figure 2:**
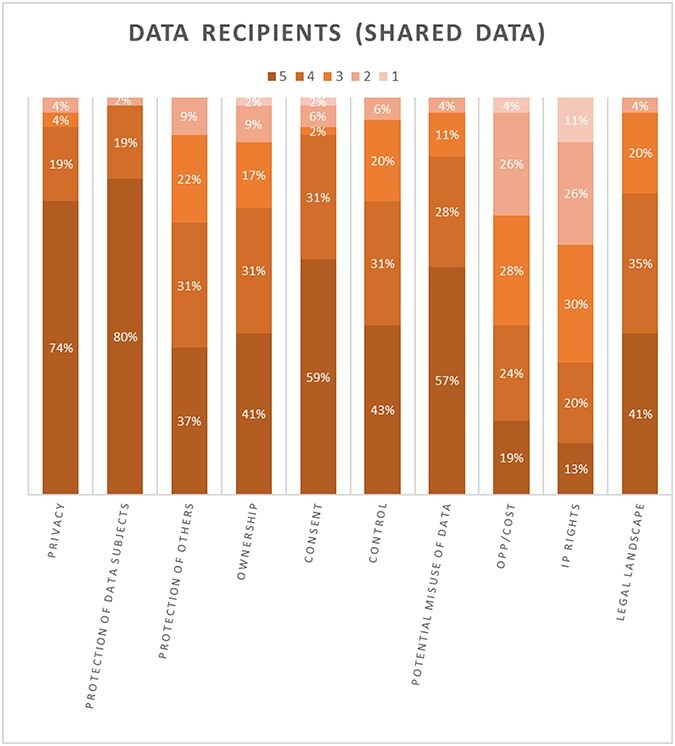
Perceived Significance (Data Receiver)

In this section, we summarize the results of the five-degree-significance test, before moving to discussion of our findings from the qualitative analysis in Section B. This quantitative analysis is presented in 2 figures that show stacked column charts with 10 bars of equal size representing 10 of the barriers to data sharing that were identified in the theoretical section. Each bar shows five shades of significance (dark representing the most significant to light for the least significant) and a percentage of votes. We would urge some caution around the statistical significance of these charts given the numbers of respondents; we present these only to illustrate outliers across all 10 constructs (by reason of either high or low percentages of perceived significance). Figure 1 shows participants’ ratings when they had collected and curated the datasets themselves (i.e. ‘data holder’), while Figure 2 shows the same ratings when the datasets with which the participants were dealing had been collected by another source and then shared with them (i.e. the participant was the ‘data recipient’).

Across both figures all of the constructs have three or more shades of color, indicating that participants had different perceptions of each construct. This was perhaps to be expected given that we had explicitly sought responses from a wide range of health ecosystems. Furthermore, we note some broad patterns *across both figures*, regardless of whether the participants were responding as data holders or data receivers:

- The bottom three constructs that were overall most commonly either ranked as ‘not at all significant’ or of ‘low significance’ in the context of decisions around sharing health data (across both figures) were: Intellectual Property Rights, Opportunity Costs (e.g., time invested in data) and Ownership. It is also of note that the significance of each of these is contested, in that while some (albeit few) participants perceive them as very highly significant, others feel they are less so or not at all significant.
- The three constructs most commonly rated to be of ‘very high’ or ‘high’ significance across both figures were the Protection of Data Subjects, Privacy and the Potential Misuse of Data.

More specifically, when looking at the role of ‘Ownership’ for data holders in particular, only 22% of participants ranked ownership as highly significant when making decisions about holding and sharing health-related data (Figure 1). The only construct that ranked lower than this was ‘Intellectual Property Rights’ (which 7% of data holders consider to be highly significant). Ownership was ranked alongside ‘Opportunity Costs’, which was also felt to be highly significant by 22% of participants. Turning to data receivers, who are negotiating access to health-related data sets that others hold (Figure 2), these three constructs fare only slightly better and remain at the bottom of the scales of significance.

Conversely, the ‘Protection of data subjects’ was consistently ranked at the top of the scale of significance. This was the case for data receivers (Figure 2) where a majority of participants considered this to be of very high significance (80%); no participants ranked this as being of low significance or not significant. ‘Privacy’ was also ranked as highly significant (74%). A similar picture was presented for data holders (Figure 1) where the ‘Protection of data subjects’ again ranked first in terms of ‘high significance’ (83%), just ahead of ‘Privacy’ (69%).

Even though these differences in perceptions underline the need to be circumspect when seeking to identify a consistent narrative that underpins why stakeholders do, or do not, share health data, these results suggest that ownership, and some of the constructs most strongly associated with this—such as ‘Intellectual Property Rights’ and ‘Opportunity Costs’—are perceived by participants to be some of the least relevant in play. Conversely, participants find the protection of data subjects and privacy to rank much higher in terms of their significance. One interpretation that could follow from this is that an overt appeal to a concept of ownership does not feature in data sharing decisions, either as a barrier or otherwise, and should be discounted as a factor. However, this does not align with the persistence of ownership as a narrative in the literature, albeit one that is patchy and underdeveloped.

Further, while in the questionnaire results there was more consensus around, for example, the significance of the protection of data subjects, it was also notable that ownership (and some of the concepts that sit alongside this such as ‘Intellectual Property Rights’ or ‘Opportunity Costs’, and of control and reward) are far more contested. It is not clear why ownership and associated constructs gave rise to these marked differences of opinion, while the protection of data subjects should rank so highly. These were matters we were able to explore further in our qualitative analysis of the dataset collected from the free text questionnaire answers, and from the focus groups and interviews.

### IV.B. Competing Narratives

In analyzing the corpus of qualitative data gathered in the open answers, focus groups and interviews, we introduced and refined broad codes that aligned with the 10 constructs identified in the questionnaire. We added extracts to these existing codes when we found that participants directly substantiated one of these constructs. However, we went beyond this and created new categories and relationships to capture additional descriptions and dimensions when the data did not fit with any of the constructs; whenever the text helped clarify one particular construct, it was added to that category. Overall, open coding, in this way, refined our understanding of existing constructs but also, and crucially, allowed us to identify emerging themes or narratives. Here we present three key narratives that seem to be on-going within health ecosystems.

#### IV.B.i. Narrative One: Data protection above ownership

A key narrative is not about ownership, but about data protection and privacy. ‘Data sharing’ is not seen as a term of art encompassing a set of ideals that necessarily make research beneficial; instead it is represented as a service, or a product that is already out there, and there is evidence that it is increasingly difficult to assess the ‘quality’ of data and to know where it comes from (P12a, P6, P21). Therefore, data protection is understood as a condition of use (i.e. that the data have been gathered under a certain set of conditions and these must be respected) and a ‘hygiene factor’ (i.e. that it produces data with a clean provenance) (P5, P38). Data protection is also tightly bundled with the protection of patients’ privacy (P1, P5, P6, P12b, P12x, P16, P22, P34), translated into rules and policies that ‘prevent potential issues that may affect subjects at the emotional or physical level’ (P1) and that ensure ‘comfortable’ data sharing for all involved parties (P1).

Following the public backlash against the controversial *care.data* initiative in NHS England,^59^ this had led to increasing views that data and privacy protection is a measure of ‘trust’ and of the pressing urge to reinstate public credibility in research and healthcare institutions (P7); this situation raises an interesting point of tension between sharing and withholding data, as this participant puts it:

‘… There is a tension between the expectations from the funders and everybody else who want to share data, and the expectations of the information governance committees (IGCs) (I am talking here about NHS Digital and even SAIL) ...who expect you to be very careful with the way you are sharing data because of confidentiality issues and data protection. IGCs are torn between allowing total open access, [-i.e.] people storing data in archives and having the whole community downloading data for free, and enforcing data protection… [because] there is no such a thing as an anonymized data and if you have an intruder dataset you can identify the person… *but what they are really afraid of is that the Daily Mail suddenly publishing data on that one person that is identified*, …’ (P12b) [emphasis added].

Public trust and perception of the extent to which individuals’ data and privacy are protected is therefore pivotal to enable sharing processes. Participants agreed that this is the real barrier to data sharing, in contrast to data and privacy protection as a legal concept and as an operational feature of the conditions of data transfers:

‘In the regular patient care in particular, no-one talks about ownership, only protecting the data… Once [we] start reusing the data, … then suddenly we have an anonymous data set that the clinic sells, or that we share with the University… but then we make a data transfer agreement in which we say, we give you data, but you give us something back: in that data transfer agreement, we demand back, for instance, authorships on the publications that they do on ‘our data’. Of course we also make rules where we make sure that we kind of push our stewardship to them, so we also say that you have to be a good steward, so you have to delete the data when we ask, and you have to protect it and secure it, but we also ask something back, so it’s more than the stewardship, right, because if we saw ourselves pure as stewards - here’s the data, you have to take care of that - if we ask something back it means that (pause)’ (P12a).

Thus, this first observation suggests that the privacy of the participants - secured and manifest through their data protection rights - is the dominant concern to researchers, and is a dominant way of discussing the relationship of researchers to the data in relation to data sharing.

#### IV.B.ii. Narrative Two: Ownership through the relationship process of data sharing

From the two previous quotes, it also appears that ownership is a concept that is not commonly used or seen to be relevant in data sharing. The focus is, instead, on matters such as working to secure the dataset, guarantee its integrity and establish sufficient anonymity. The priority is to warrant the quality of the datasets and the use of best practices so that data subjects are protected and public trust is maintained. However, the argument of the participant in the immediate last quote goes beyond this point. The quote reveals a dualism in the approach to ownership as a term and as a practice. Initially, the participant discusses the absence of a context for ownership, but then describes the use of agreements that warrant the satisfaction of the parties: ownership is materialized in action and through controls over data exchange. The possibility of having a formal data agreement and reuse policy is arguably a concretization of ownership through an institutional and legal framework, which enables the circulation of data by facilitating and circumscribing their use.

This is intriguing, given our initial interest in this research: we knew that appeals to ownership are persistent, and we knew that this was not on the basis of the legal literature review alone. When we asked participants directly if ownership is a barrier to data sharing, many answered that ownership ‘does not exist because data… just travel endlessly’ (P12a, P12b, P12x, P31, P6, P38). One participant argued that ‘ownership is a hard word’ because it has ‘this property association’ (P12a).

A dataset, however, is seen as labor; it is, among other things, the work undertaken to collect, curate and perform responsible stewardship, all of which may lead someone to refer to a dataset as ‘my data’. In other words, producing research data is inherently a transactional mode that often starts with funding and is characterized by known rules of engagement among involved parties. These consist first in identifying sides in the transaction (P12a); so, if the data are shared among academic partners, university-based academic partnerships become the standard mode of governance. Otherwise, any commercial use of research data should then demand monetary compensation in order to redeem the cost of data stewardship (P12a, P12b, P12x). As one participant puts it:

‘[we] take 3 or 3.5 years of a Ph.D. time to build the data and 0.5 in analysis/publication before [we] put data out. This is not sustainable if [we] cannot sell data at all… so we demand something back: for instance, authorship on the publications that they do on ‘our’ data, or a monetary value (we can get, I do not know, €50,000 for 2,000 patients…)’ (P12x).

In both of these cases, however, ownership of data is transient; it is instantiated through the act of creating a data use agreement or a data transfer agreement. This enables the data holder to place conditions on the data seeker, perhaps to pay for access but also by asking them to protect, secure and delete the data after the stated purposes of access have been fulfilled. Data seekers’ proposals, i.e. what they plan to do with the data, also influence the outcome of the exchange. For example, a ‘repository function’ could be required to reduce data breaches using trust via certification and training (P12x). In cancer research, for instance, data could be acquired with a time-limited embargo on publications, or on research use only (P12x). Thus, many of the features of ownership, as it is commonly understood in lay terms, appear within these transactional behaviors: control, payment, conditions of use, etc. It makes sense, then, that ownership is a convenient shorthand; but it does not adequately capture the competing narratives between narrative two and narrative one, i.e. when transactions must also deliver protections for data subjects.

#### IV.B.iii. Narrative Three: Stewardship versus ownership

Because of the sensitive nature of personal data and the requirements of data and privacy protection, the data holder keeps an important role in ensuring stewardship and in interpreting the requirements for data use (once the data are shared and in the hands of third parties). This challenges the use of ‘ownership’ (at least in its colloquial sense) as an appropriate term:

‘…we [researchers] shift datasets around all the time, from the referring hospital we get a lot of data…so that we can treat the patient and we send the data back and… *these infrastructures never talk about ownership, only security and stewardship*’ (P12a) [emphasis added].

Stewardship was an emerging code that we did not use in the constructs in the questionnaire. Stewardship is conceptually different from ownership: it provides data holders with a duty to require sufficient controls over the data to warrant its protection in the construction of data sharing. In other words, stewardship puts the responsibility of preventing breaches, hacks and malicious misuse (P12a) on the data holder, and forces them to design their own checks and protocols to reach through the transfer of data to third parties. This could lead to non-standard and sometimes lengthy access and compliance procedures, and hence could become a barrier to data sharing (P17).

Stewardship is essential to a transactional mode of data production and repurposing; however, stewardship is not discussed as a concept, but as a set of practices enabling data to remain fluid and re-purposable (P12a, P12x). These include both emerging codes, such as data fluidity, best practices, quality, security, controls, anonymity, as well as existing codes, such as consent, control, etc.

The negotiation of this practical stewardship is, therefore, very interesting. Much of this is negotiation between parties, but there are also institutions involved in the framing of stewardship.^60^ Information Governance Committees (IGCs), such as NWIS, Generation Scotland, NHS Digital and other Health Informatics institutions are important political and governance actors (P12a). Their role consists primarily in defining the technologies of stewardship, but they also run data linking initiatives, so they are also data holders. However:

‘…these processes of linking data can still be bureaucratic with no distinction made between consented and unconsented datasets… [so that IGCs] could still erect barriers… [and] justify a decision not to share data… Processes are gradually evolving but there still needs to [be] greater standardization… and there are still inconsistencies between its constituent parts and also variable application of principles relating to IG’ (P17).

Accordingly, IGCs are sometimes depicted as inefficient organizations, and their models of stewardship are seen as the real barrier. This relies on the implementation of a legalistic vocabulary of use agreements and compliance that leads to massive bureaucratization, paperwork and delays (P1, P21, P29).

## V. DISCUSSION AND CONCLUSIONS

Our initial review of the law and literature confirmed that while those who hold, use, and share health-related data might reasonably be concerned to ensure that they do not fall foul of data protection and confidentiality requirements, this does not account for the persistence of notions of ownership, per se. We have therefore suggested that, in order to explore notions of ownership fully in this environment, it is unrealistic to discount the power of these proprietary feelings and attitudes towards ‘my data’; ‘ownership’ used as a colloquial and metaphorical concept is prevalent in the data sharing community.

To discount the shorthand of ‘my data’ is to fail to take into account the different ways in which stakeholders use and understand their relationships to data.^61^ It also confuses appeals to ownership that might be based in a desire for due recognition with those designed to close down sharing or further use. Furthermore, multiple actors and tasks are involved and might include: individuals sharing data; researchers working on, curating and aggregating data; and/or researchers seeking access to data. Revealing this complexity under the single umbrella term of ‘my data’ suggests that there may be competing narratives towards and around data sharing, depending on the actors involved and the tasks they are undertaking. Thus, we propose a holistic approach to notions of ownership that is able to account for these broader considerations and competing narratives. Put simply: ‘ownership’ does not mean one static thing to all who deploy the term. This research captures this and reveals more of what is behind the label.

Our research shows that there is a range of perceptions of legitimate interests in data sharing. A major concern is about the protection of the rights and interests of data subjects. This meta obligation reaches across data ecosystems and through data sharing agreements. Moreover, the specific transactional obligations that are then imposed in any given data sharing agreement are undoubtedly seen as barriers to data sharing, but they were not often expressed as a negative barrier; data protection and privacy were expressed as necessary and ‘positive barriers’ in data sharing, positive in that they are concerned with demonstrating respect and trustworthiness and in building confidence in data sharing. On one level, this can be seen as simply reflecting the understanding of the research community about its obligations in relation to personal data. However, the property lawyer might become interested: the idea of reach-through consent conditions begins to sound very similar to the property concept of ‘nemo dat quod non habet’—you cannot give what you do not have. If personal data in data sets are ‘things’ for the purpose of this discussion, the transfer of those things is about the relationships between people about those things, and this ostensibly puts us squarely in the realm of property theory. However, is it a useful thought or a helpful place to be? Perhaps, in the sense that property law has had to think about how to attach limitations on the use of shared things (property), and has developed, for example, registration schemes. Thus, regardless of whether there is property in personal data as a matter of strict law, there are analogies to be explored. Equally, the limits of the property analogy must be recognized, for example, when narrative one suggests that the overarching concern is seeking to protect data subjects and not to stake any property claim per se.

A further important concern is the quality of the data. Participants indicated that a barrier to data sharing was a concern about the quality of data that were being received, and about what would be done with data that were shared. This was again a concern for legitimate interests—here, the public interests in maintaining high standards and quality in science. One key aspect of addressing this, and which was discussed, is the cost of properly curating data. Participants indicated that ensuring the quality of research data, including the meta data, has high cost, and if society wishes to embrace open access and data sharing, this cost must be met. Narrative two reveals this, and suggests that this might compete with narrative one, if only in the sense that recovering costs while making data more open now must be added to the foundational obligations of ensuring enduring protection of data subjects' interests.

Few of the participants talked directly about the monetary value of data. When it was raised, it was in the context of data generated in hospitals, and was part of a broader understanding of the (need for) commercial considerations in that setting. However, many participants spoke about other rewards for the labor associated with data. Primarily, this was articulated in relation to publications. Again, a property theorist might feel very much at home in this discussion. Property, especially IP, is often discussed in relation to Locke’s theory of property: property is gained through adding value to a resource through labor. This was another legitimate barrier to data sharing—that those who add value to data through their labor, by generating data or analyzing data, should be given the opportunity to benefit from that effort. It is clear that actors at different points in the data sharing chain have different perceptions of value and access, but this is part of the broad discussion of ‘ownership’ or ‘stewardship’ of data. But stewardship and ownership are different in a fundamental respect: while ownership in a strict sense permits the act of ‘doing nothing’ as an option because final control rests with the putative owner, stewardship is concerned both with protecting an artifact and promoting its responsible use. In this important sense, then, stewardship can be seen as a further necessary and ‘positive’ barrier to data sharing.

These findings have a number of implications for data governance more widely. First, they suggest that because stakeholders do not have a unitary view of what ‘ownership’ involves this is not a good foundation for governance at present without further clarification. Multiple understandings—or competing narratives, as we have described them above—of ownership are unlikely to promote clear communication across stakeholders. However, it is equally not the case that the concept is without use. What is also clear is that the concept of a barrier is also neither obvious nor unitary. The work showed that there are barriers to data sharing, certainly, but that there are legitimate barriers to data sharing, and the governance structures created to develop data sharing have to recognize these. It is particularly interesting to consider how the language of ‘stewardship’, unlike ‘ownership’, has traction in the discussion. As indicated above, the terms are not synonymous, and stewardship might be a conceptual vehicle that could accommodate the issues raised by the conversation about the meaning of ‘my data’. Certainly, there is a legitimate counterpoint to a call for unconditional ‘open access’ to research data, particularly in health data research.

In sum, what this research has shown is that there is indeed much power in the social imaginary of ownership with respect to data in the biomedical sphere; however, that power emerges and is enacted in unexpected ways by multiple actors within the ecosystem, often in ways driven by competing narratives about what is at stake. Importantly, formal legal property-type appeals to ownership appear to have far less power in the stories that are told about data than the ethical and social concerns that underpin responsible biomedical research.

## Footnotes

1 Patty Kostkova et al., *Who Owns the Data? Open Data for Healthcare*, 4 Front. Public Health (2016), doi: 10.3389/fpubh.2016.00007

2 The Royal Society, *Science as an Open Enterprise* (2012), p7, https://royalsociety.org/topics-policy/projects/science-public-enterprise/report/ (accessed June 11, 2020).

3 Lisa M. Federer et al., *Biomedical Data Sharing and Reuse: Attitudes and Practices of Clinical and Science Research Staff*, 10(6) Plos One 1 (2015), at 2 doi: 10.1371/journal.pone.0129506.

4 The Data Harvest Report, *Sharing Data for Knowledge, Jobs and Growth* (2014), https://www.rd-alliance.org/data-harvest-report-sharing-data-knowledge-jobs-and-growth.html (accessed June 11, 2020).

5 *The Four Signatories to the Concordant on Open Data Research Are HEFCE, Research Councils UK, Universities UK and Wellcome*  https://www.ukri.org/files/legacy/documents/concordatonopenresearchdata-pdf/ (accessed June 11, 2020).

6 *Concordant on Open Data Research* (2016), https://www.ukri.org/files/legacy/documents/concordatonopenresearchdata-pdf/ (accessed June 11, 2020).

7 Federer et al., *supra* note 3.

8 Elizabeth Pisani and Carla AbouZahr, *Sharing Health Data: Good Intentions Are Not Enough*, 88(6) Bulletin of the World Health Organization, 462–466, 462 (2010) doi: 10.2471/BLT.09.074393.

9 *Id*. at 462–466.

10 Bartha M. Knoppers et al., *A Human Rights Approach to an International Code of Conduct for Genomic and Clinical Data Sharing*, 133(7) Hum. Genet. 895 (2014) doi: 10.1007/s00439-014-1432-6. See also the work of the Global Alliance for Genomics and Health on accommodating diversity in data sharing and use: https://www.ga4gh.org (accessed June 11, 2020).

11 This research project was commissioned and funded by Wellcome’s Expert Advisory Group on Data Access (EAGDA). The *DataTerms* fieldwork was undertaken under approval from the Edinburgh University Law School Research Ethics Committee; participation was on the basis of informed consent.

12 The Royal Society and The British Academy, *Data Management and Use: Governance in the 21st Century* (2017), https://royalsociety.org/-/media/policy/projects/data-governance/data-management-governance.pdf (accessed June 11, 2020).

13 *Id*. at 15.

14 *Id*. at 6 and 29. Prainsack explores this developing tension in the conceptualization of property in relation to data in Barbara Prainsack, *Logged Out: Ownership, Exclusion and Public Value in the Digital Data and Information Commons*, 6(1) Big Data and Society 1 (2017) doi: 10.1177/2053951719829773. She shows the tension between (what we would understand to be) commodity-reward and dignity-safeguarding concepts of property, and within safeguarding, the nature of commons conceptualizations.

15 Knoppers et al., (2014), *supra* note 10.

16 Edward S. Dove, *Biobanks, Data Sharing, and the Drive for a Global Privacy Governance Framework*, 43(4) J. Law Med. Ethics 675 (2015) doi: 10.1111/jlme.12311.

17 Barbara E. Bierer, Mercè Crosas and Heather H. Pierce, *Data Authorship as an Incentive to Data Sharing*, 376 New Eng. J. Med. 1684 (2017) doi: 10.1056/NEJMsb1616595.

18 The CODATA Report, *infra* note 37.

19 The Royal Society, *supra* note 2.

20 Mariarosaria Taddeo, *Data Philanthropy and Individual Rights*, 27 Minds and Machines 1–5, 2 (2017)

21 *Id*.

22 Willem G. van Panhuis et al., *A Systematic Review of Barriers to Data Sharing in Public Health*, 14 Bmc Pub. Health 1 (2014). At p. 2, the authors suggest that: ‘Unless these barriers are better understood, solutions may remain ineffective.’

23 *Id.* at 5–6.

24
*Id.*

25 *Id.* at 5.

26 *Id.* at 5.

27 *Id.* at 5.

28 Michael Edelstein and Jussi Sane, *Overcoming Barriers to Data Sharing in Public Health: A Global Perspective*, Chatham House, The Royal Institute of International Affairs 1 (2015).

29 *Id.* at 8.

30 *Id.* at 8.

31 General Data Protection Regulation (EU) 2016/679 (OJ L 119, 4.5.2016, p. 1–88).

32 Now encompassed by Article 8 of the European Convention on Human Rights.

33 World Intellectual Property Organisation, http://www.wipo.int/about-ip/en/ (accessed June 11, 2020). This is also the definition used in the CODATA Report, note 37, at p. 36.

34 Dave A. Chokshi, Michael Parker and Dominic P. Kwiatkowski, *Data Sharing and Intellectual Property in a Genomic Epidemiology Network: Policies for Large-Scale Research Collaboration*, 84 Bull. World Health Org. 382 (2006).

35 Pamela Andanda, *Managing Intellectual Property Rights Over Clinical Trial Data to Promote Access and Benefit Sharing in Public Health*, 44 IIC 140 (2013), at 141 doi: 10.1007/s40319-012-0016-z.

36 *Id.* at 141, see footnote 6, citing E. A. de Carvalho et al., *Application Description and Policy Model in Collaborative Environment for Sharing of Information on Epidemiological and Clinical Research Data Sets*, 5 Plos One (2010).

37 RDA-CODATA, *Legal interoperability of research data: principles and implementation guidelines*, October 2016, http://www.codata.org/uploads/Legal%20Interoperability%20Principles%20and%20Implementation%20Guidelines_Final2.pdf (accessed June 11, 2020), referred to as ‘The CODATA Report’.

38 *Id*. at 1. The report explicitly carves out, for example, consideration of other legal matters that may impact on data sharing, such as ‘human privacy’, as well as other perspectives beyond the purely legal.

39 *Id*. at 8.

40 *Id*. at 19.

41 *Id*. at 9.

42 Panhuis et al., *supra* note 22, at 5.

43 Nayha Sethi and Graeme T. Laurie, *Delivering Proportionate Governance in the Era of eHealth*, 13 Med. Law Int. 168 (2013).

44
*Id.*

45 Jonathan Montgomery, *Data Sharing and the Idea of Ownership*, 23 The New Bioethics 81 (2017).

46 Knoppers et al., *supra* note 10, at 897.

47 Panhuis et al., *supra* note 22, at 4.

48 Lisa M. Federer et al., *Biomedical Data Sharing and Reuse: Attitudes and Practices of Clinical and Science Research Staff*, 10 Plos One 1 (2015).

49 *Id*. at 13.

50 *Id.* at 2.

51 *Id.* at 14.

52 *Id.* at 14.

53 M. Martin et al. eds., Readings in the Philosophy of Social Science (1994).

54 Barbara Czarniawska, Narratives in Social Science Research (2004).

55 Margarete Sandelowski, *Telling Stories: Narrative Approaches in Qualitative Research*, 23 Image 161 (1991). doi:10.1111/j.1547-5069.1991.tb00662.x.

56 Vito De Lucia, *Competing Narratives and Complex Genealogies: The Ecosystem Approach in International Environmental Law*, 27 J. Env. Law 91 doi: 10.1093/jel/equ031; Patrick Dawson and David Buchanan, *The Way It Really Happened: Competing Narratives in the Political Process of Technological Change*, 58(7) J. Hum. Rel. 845 (2005) doi: 10.1177/0018726705057807.

57
*Id.*

58 Dawson and Buchanan, *supra* note 56.

59 The Caldicott Review 2016, http://www.geneticalliance.org.uk/news-events/news/data-security-consent-and-opt-outs-in-the-nhs-and-social-care-the-caldicott-review/.

60 Graeme T. Laurie et al., *Charting Regulatory Stewardship in Health Research: Making the Invisible Visible?* 27 Cam. Quar. Heal. Ethics 333 (2018).

61 Alexander Morgan Capron et al., *Ethical Norms and International Governance of Genetic Databases and Biobanks: Findings from an International Study*, 19 Kenn. Inst. Ethics J. 101 (2009).

